# Adverse Events and Measurement of Dissociation After the First Dose of Esketamine in Patients With TRD

**DOI:** 10.1093/ijnp/pyac081

**Published:** 2022-12-16

**Authors:** David Williamson, Ibrahim Turkoz, Ewa Wajs, Jaskaran B Singh, Stephane Borentain, Wayne C Drevets

**Affiliations:** Janssen Scientific Affairs, LLC, Titusville, New Jersey, USA; Department of Psychiatry and Health Behavior at Augusta University, Augusta, Georgia, USA; Department of Statistics and Decision Sciences, Janssen Research & Development, LLC, Titusville, New Jersey, USA; Department of Neuroscience, Janssen Research & Development Belgium, Beerse, Belgium; Neurocrine Biosciences, San Diego, California, USA; Department of Neuroscience, Janssen Research & Development, LLC, San Diego, California, USA; Department of Global Medical Affairs, Janssen Research & Development LLC, Titusville, New Jersey, USA; Department of Neuroscience, Janssen Research & Development, LLC, San Diego, California, USA

**Keywords:** esketamine, CADSS, dissociation, BPRS+, psychosis

## Abstract

**Background:**

“Dissociation” comprises distinct phenomena, some of which are associated with esketamine treatment and some may overlap with positive symptoms of psychosis. Relationships between dissociation and psychotic symptoms assessed by ­clinician report vs conventional rating scales were investigated in a post hoc analysis of data from the initial treatment session in an ­open-label, ­long-term safety, phase 3 study of esketamine plus a newly initiated oral antidepressant in patients with treatment-resistant depression.

**Methods:**

Adverse events of dissociation or psychosis were examined via investigator report and the Clinician Administered Dissociative States Scale (CADSS) and Brief Psychiatric Rating Scale-Plus, respectively, 40 minutes post first esketamine dose. The range of CADSS total scores associated with investigator-reported severity of dissociation was determined by equipercentile linking. Logistic regression models and receiver operating curve analysis explored the CADSS cutoff point for determining presence/absence of dissociation. Frequency of response to specific CADSS items was examined to investigate qualitative differences in the pattern of symptoms reported across investigator-reported levels of adverse event severity.

**Results:**

Dissociation was reported as an adverse event in 14.3% (109/764) of patients. Severity of most CADSS items increased with the severity of investigator-reported dissociation. No CADSS cutoff point discriminated well between the presence and absence of dissociation events. Hallucinations were reported as adverse events in 5 patients; none reported delusions.

**Conclusions:**

CADSS scores and severity of dissociation adverse events move generally in the same direction; however, there is substantial variability in this relationship. No signature profile of dissociative experiences was revealed, and psychotic symptoms were uncommon.

**Trial Registration:**

Clinical Trials.gov identifier: NCT02497287

**Significance Statement**

Transient dissociation has been reported with esketamine treatment. Results of a post hoc analysis presented here further characterize the nature and severity of dissociation reported in an open-label phase 3 study (SUSTAIN-2) of esketamine plus a newly initiated oral antidepressant in participants with treatment-resistant depression (TRD). After the first esketamine dose, dissociation was reported as an adverse event in 14.3% (109/764) of patients. Investigators characterized most dissociation events as mild (n = 78), some as moderate (n = 26), and few (n = 5) as severe. Severity of most items of the Clinician Administered Dissociative States Scale (CADSS) increased with the severity of investigator-reported dissociation events. No CADSS cutoff point discriminated well between the presence and absence of investigator-reported dissociation adverse events. Hallucinations or delusions were reported as adverse events in 5 and 0 patients, respectively. In summary, no signature profile of dissociative experiences was revealed, and psychotic symptoms were uncommon.

## INTRODUCTION

Dissociation is a clinical construct that incorporates a variety of different types of symptoms, ranging from disturbances in perception of sensory, proprioceptive, or temporal information to disturbances in one’s sense of self or identity. The extent to which the nature of these dissociative experiences is consistent across different clinical conditions or diagnoses has received relatively little attention. For instance, a recent meta-analysis of clinical and nonclinical samples examining the relationship between dissociation and psychotic symptoms in patients with psychotic disorders found a “robust” relationship between dissociative experiences and multiple positive psychotic symptoms but less consistent relationships with negative symptoms (Longden et al., 2020). However, the research reviewed by this meta-analysis did not include dissociation associated with specific pharmacological interventions, such as those reported in studies using ketamine or esketamine to treat major depressive disorder (MDD); in these investigations, rates of positive psychotic symptoms have been very low to absent (Daly et al., 2019; Popova et al., 2019) despite encountering relatively high rates of dissociative adverse events.

The patterns of dissociation-related symptoms that occur in association with i.v. ketamine treatment as quantified by the Clinician-Administered Dissociative States Scale (CADSS) have been described by multiple groups (Singh et al., 2016; Niciu et al., 2018; van Schalkwyk et al., 2018). Using confirmatory factor analysis (CFA) for ordered variables, Niciu and colleagues (2018) reported that the CADSS responses of a sample of 126 adults with treatment-resistant depression (TRD) (associated with MDD or bipolar disorder) sorted into 3 factors, similar to those proposed by Bremner and associates (1998) in their initial scale development for patients with post-traumatic stress disorder. In contrast, using exploratory factor analysis with oblique rotation, van Schalkwyk and colleagues (2018) found a single-factor solution to be most appropriate in their sample of 110 patients with TRD receiving their first dose of i.v. ketamine. In both groups, measurement was performed 40 minutes post dose, the time point at which maximal dissociative effects were observed.

It has also been reported that the correspondence between a clinician’s perception of dissociation occurring as an adverse event vs dissociation as characterized by structured measurements (e.g., the CADSS) may vary (Acevedo-Diaz et al., 2020a). This is particularly relevant within the context of clinical trials, wherein dissociation-related adverse events are represented in product labeling that will inform clinicians’ and/or patients’ perspectives on the safety and tolerability of a product. In addition, the presence or absence of dissociation has been linked to the likelihood of a therapeutic response to some medications (Dakwar et al., 2014; Luckenbaugh et al., 2014; Niciu et al., 2018; Acevedo-Diaz et al., 2020b; Grabski et al., 2020), though this finding has been inconsistent across studies of ketamine or esketamine (Chen et al., 2022). Acevedo-Diaz and colleagues reported data on the relationship between CADSS scores and reported dissociation adverse events in a sample of 188 participants in 4 placebo-controlled trials of ketamine for TRD associated with either MDD or bipolar disorder (Acevedo-Diaz et al., 2020a). These authors noted that the CADSS and reported dissociation adverse events were positively related at 40 minutes post ketamine infusion, with the sum of 18 dissociative events accounting for 36% of the variance in CADSS total score. There was no reported exploration, however, of the extent to which severity of reported adverse events was related to CADSS scores, of whether specific items on the CADSS were more likely to be endorsed at different severity levels of reported events, or of the extent to which psychotic symptoms were present in patients manifesting dissociation, all of which may be relevant to clinical decision-making.

Treatment-emergent adverse events, including dissociation and psychosis, were monitored and recorded in the pivotal registration trials of esketamine, thereby providing the opportunity to evaluate the relationship between these (i.e., between patient experience and clinician-reported assessment) as well as adverse event reports of dissociation or psychosis. We thus conducted post hoc analyses of data from the SUSTAIN-2 study (Wajs et al., 2020) with the aims of determining the following: (1) What is the underlying factor structure of the CADSS in this sample? (2) What CADSS total score best discriminates patients clinically identified as experiencing an adverse event of dissociation from those not identified as experiencing dissociation? (3) What CADSS total score ranges are associated with the different severity levels of the investigator-reported adverse event of dissociation? (4) Which CADSS items are endorsed most frequently, and how do these items relate to the presence and severity of “dissociation” adverse event reports? (5) To what extent are positive psychotic symptoms associated with reported dissociation?

## MATERIALS AND METHODS

### Ethical Practices

An independent review board/ethics committees approved the SUSTAIN-2 protocol at each study site, and written informed consent was obtained from all patients before they were enrolled in the study. SUSTAIN-2 is registered at clinicaltrials.gov (identifier: NCT02497287). Study methods pertaining to the work reported herein are summarized below.

### Patients

SUSTAIN-2 enrolled adults (≥18 years old) with a diagnosis of recurrent MDD or single episode (≥2 years) MDD without psychotic features per DSM-5 criteria (American Psychiatric Association, 2013). Participants had failed to respond to an adequate course of at least 2 oral antidepressants during the current depressive episode and had a Montgomery-Åsberg Depression Rating Scale (Montgomery and Åsberg, 1979) total score ≥22 at screening. A full list of the inclusion and exclusion criteria is available elsewhere (clinicaltrials.gov: NCT02497287).

### Study Design

SUSTAIN-2 was a global, open-label, multicenter, phase 3 study of TRD that evaluated the safety and tolerability of esketamine plus a newly initiated oral antidepressant for up to 1 year (Wajs et al., 2020). The study comprised 4 phases: a 4-week screening phase, a 4-week induction phase, an up to 48-week optimization/maintenance phase, and a 4-week follow-up phase. A more complete description of study protocol and overall study results has been published (Wajs et al., 2020).

### Study Drug

During the induction phase, patients self-administered esketamine nasal spray twice a week for 4 weeks as a flexible-dose regimen, beginning at 28 mg (in those aged ≥65 years) or 56 mg. Subsequent doses could be adjusted (<65 years: 56 or 84 mg; ≥65 years: 28, 56, or 84 mg) based on efficacy and tolerability. All patients were also taking a newly initiated oral antidepressant (i.e., duloxetine, escitalopram, sertraline, or venlafaxine extended release).

### Safety Assessments

Adverse events were monitored throughout the study. Dissociative and positive psychotic symptoms, respectively, were assessed pre-dose and 40 and 90 minutes post-dose using the CADSS (Bremner et al., 1998) and the positive symptom subscale of the Brief Psychiatric Rating Scale (BPRS+) (Overall and Gorham, 1962; Ventura et al., 1993). The CADSS consists of 23 subjective items; each item is rated on a scale from 0 (not at all) to 4 (severe), with the total score ranging from 0 to 92. Developed by Ventura and colleagues (1993), the BPRS+ consists of 4 items from the BPRS (i.e., suspiciousness, hallucinations, unusual thought content, and conceptual disorganization) felt to be most closely tied to positive symptoms evident in patients with psychotic disorders. Each of these items is rated on a scale of 0 (not present) to 6 (extreme) (Ventura et al., 1993). A total score is derived by summing the individual items with a range of 0 to 24 and a higher score representing a more severe condition.

Adverse events were also assessed based on investigator report. The SUSTAIN-2 protocol specified that any untoward medical occurrence that was new in onset or increased in severity following treatment initiation should be reported as a treatment-emergent adverse event. The protocol did not provide investigators with specific guidance of what symptoms constitute an adverse event of dissociation or how/when dissociation as an adverse event should be reported.

Dissociation is a unique preferred term in the Medical Dictionary for Regulatory Activities dictionary, and most site investigators reported “dissociation, dissociative symptoms” as adverse event terms that are coded to the preferred term “dissociation.” However, site investigators also reported other terms to describe dissociative symptoms that have been coded to “dissociation” per instruction from the Food and Drug Administration, such as symptoms of auditory and visual disturbances, illusion, feeling abnormal (feeling of floating, flying, falling, detached, heaviness, lightness, etc.), and dreamy state. While potentially allowing for the possibility of increased between-site variability, this approach could be argued to provide a more generalizable picture of what may be happening across a variety of treatment settings, wherein one would not expect the level of standardization of adverse event reporting obtained by using structured psychometric instruments.

### Statistical Methods

Post hoc analyses were performed on data collected during the first esketamine treatment session. Data were analyzed from the 40-minute measurement taken on the first day of dosing, as this provided the greatest range of CADSS total scores (Niciu et al., 2018; van Schalkwyk et al., 2018).

Confirmatory factor analyses of these data were performed to determine the goodness-of-fit of recently published 1- and 3-factor solutions (Niciu et al., 2018; van Schalkwyk et al., 2018). Following the recommendations of Kline (2016), model fit was evaluated in CFA using a mix of global and local fit indices: chi square (χ^2^), residual-based measures (root mean square of approximation [RMSEA]), standardized root mean square residual (SRMR), and an index assessing incremental goodness-of-fit (Comparative Fix Index [CFI]). Given our sample size (n > 200), χ^2^ is viewed as problematic for evaluating model fit because the test statistic is more likely to signal a significant difference between distributions for larger samples (Hu and Butler, 1999). However, the relative χ^2^, calculated as the χ^2^ statistic divided by degrees of freedom, has been found to mitigate this issue to some extent, with the target value being between 2 and 5 (Wheaton et al., 1977; Kline 2016). RMSEA is an absolute fit index that examines the discrepancy between the hypothesized model and an optimal model. Values closer to zero indicate better fit (Xia and Yang, 2019), with “acceptable” values being <0.08 (Fabrigar et al., 1999). The SRMR also examines fit between the hypothesized model and the sample covariance matrix, with a desirable result being <0.08. In contrast, CFI examines the discrepancy between the data and a baseline model (i.e., a model with the worst fit), while adjusting for sample size. Values range from 0 to 1, with higher values indicating a better fit. Values for acceptable models generally are ≥0.90.

As neither the 1-factor nor 3-factor solutions proved satisfactory (data reported in Results section), an exploratory factor analysis using principal axis factoring and direct oblimin rotation was conducted to identify the underlying structure of CADSS items. The oblique direct oblimin rotation, appropriate for correlated factors, was used due to the inherent correlation often observed between psychiatric symptoms (Farbrigar et al., 1999; Peralta and Cuesta, 2001; Russell, 2002). The number of factors was determined by examining the scree test, eigenvalues, simple structure, and clinical interpretability of the resulting factors.

Frequency counts of the CADSS items and the BPRS+ total scores were stratified according to investigator-reported severity of dissociation (i.e., “not reported,” “mild,” “moderate,” or “severe”). Given the skewed nature of the data, with most patients having low scores on the BPRS+ and CADSS, a Spearman rank correlation was performed to determine the relationship between the measures.

To investigate the relationship between CADSS scores and investigator-reported severity of dissociation, equipercentile linking following the method of Leucht (2005) was used to determine the range of CADSS scores associated with reported severity level. Similarly, we investigated the relationship between the reported presence of a dissociation treatment-emergent adverse event (as defined by an adverse event report of mild, moderate, or severe severity) with the presence of a dissociation event according to the Food and Drug Administration–agreed upon criterion of a CADSS total score >4 (Bremner, 2014). In addition, presence of a dissociation treatment-emergent adverse event (as defined by an adverse event report of mild, moderate, or severe severity arising during treatment that was not present prior to treatment) and the absence of such events were mapped to a range of CADSS total scores to capture sensitivity (true positive rate) and specificity (true negative rate) for each CADSS total score vs incidence of treatment-emergent adverse events. The Youden Index (1950), which balances sensitivity and specificity, was utilized to identify the CADSS total score that discriminates between those classified as experiencing dissociation per adverse event report and those who were not. Logistic regression models along with receiver operator curve analysis were used to identify the optimal CADSS cutoff for determining the presence or absence of an investigator-reported adverse event of dissociation, at any level of severity.

### STUDY RESULTS

A total of 764 patients with TRD were included in the post hoc analyses (Table 1). Their mean (SD) age at the time of MDD diagnosis and at the time of study enrollment was 35.3 (13.7) and 51.5 (13.6) years, respectively. Approximately two-thirds (62.6%) were female. The majority (85.0%) was White.

**Table 1.** Demographics and Clinical Characteristics

	Adverse events of dissociation				Total N = 764
	Not reported n = 655	Mild n = 78	Moderate n = 26	Severe n = 5	
Age (y), mean (SD)	51.9 (13.7)	49.5 (13.6)	48.4 (11.1)	44.6 (10.6)	51.5 (13.6)
Sex, n (%)					
Male	236 (36.0)	40 (51.3)	10 (38.5)	1 (20.0)	287 (37.6)
Female	419 (64.0)	38 (48.7)	16 (61.5)	4 (80.0)	477 (62.4)
Race, n (%)					
Asian	69 (10.5)	9 (11.5)	1 (3.9)	1 (20.0)	80 (10.5)
Black or African American	14 (2.1)	1 (1.3)	0	0	15 (2.0)
White	553 (84.4)	67 (85.9)	25 (96.2)	4 (80.0)	649 (85.0)
Other/multiple/not reported	19 (2.9)	1 (1.3)	0	0	20 (2.6)
Oral antidepressant, n (%)					
Duloxetine	213 (32.6)	19 (24.4)	10 (38.5)	1 (20.0)	243 (31.9)
Escitalopram	173 (26.5)	36 (46.2)	9 (34.6)	3 (60.0)	221 (29.0)
Sertraline	130 (19.9)	17 (21.8)	3 (11.5)	1 (20.0)	151 (19.8)
Venlafaxine XR	138 (21.1)	6 (7.7)	4 (15.4)	0	148 (19.4)
Age when diagnosed with MDD (y), mean (SD)	36.0 (13.9)	32.8 (11.5)	28.7 (11.0)	24.0 (5.4)	35.3 (13.7)
Baseline MADRS total score, mean (SD)	31.3 (5.3)	30.7 (5.2)	31.4 (4.6)	28.4 (2.9)	31.2 (5.3)

Abbreviations: MADRS, Montgomery and Åsberg Depression Rating Scale; MDD, major depressive disorder; XR, extended release.

On day 1, investigators reported an adverse event of dissociation for 14.3% (109/764) of the patients. Most of the dissociation events were characterized by the investigator as mild (n = 78), some were characterized as moderate (n = 26), and few (n = 5) were characterized as severe. Severe adverse events of dissociation were more often reported for female patients and patients who were younger, on average, than those for whom mild-to-moderate dissociation events were reported, though this pattern cannot be generalized due to the small number of patients in this subgroup. Dissociative symptoms generally resolved by 1.5 hours after esketamine dosing.

Hallucinations, in contrast, were less frequent and delusions were not reported as adverse events for any patient. Hallucinations were reported in <1% of the sample (5/764). Using BPRS+ score ranges as criteria (Ventura et al., 1993), positive psychotic symptomatology was of moderate severity (BPRS+ >2 and ≤9) in 34 patients and severe in 5 patients (BPRS+ >9). Notably, of the 5 patients for whom hallucinations were reported, 2 had BPRS+ scores in the moderate range (3–9), 3 had scores in the mild range (<3), and none had scores in the severe range.

### CADSS Cutoff for Determination of Dissociation

A CADSS total score >4 was determined to be the most useful in discriminating between those who were classified as experiencing dissociation per adverse event report and those who were not, per the Youden Index (1950) (Table 2). According to this cutoff, 348 patients (45.5%) could be considered as experiencing significant dissociative symptoms. That said, this value did not discriminate groups according to whether dissociation was reported as an adverse event. Specifically, of the 109 patients for whom dissociation was reported as an adverse event, 82 (75% of these) would be detected by this cutoff. However, of the 655 patients for whom dissociation was not reported as an adverse event, 265 (40%) would be categorized as experiencing significant dissociative symptoms by this CADSS threshold. Thus, this cutoff is characterized by moderate sensitivity but poor specificity.

**Table 2.** Diagnostic Characteristics of CADSS When Used to Identify Investigator-Reported Dissociation Adverse Events^*a*^

Test-positive criteria on CADSS	Sensitivity	Specificity	Youden Index: Sensitivity + Specificity −1
0+	1.000	0.000	0.000
1+	0.954	0.273	0.227
2+	0.908	0.385	0.293
3+	0.862	0.467	0.330
4+	0.798	0.527	0.325
5+	0.752	0.594	0.346
6+	0.697	0.640	0.337
7+	0.642	0.667	0.309
8+	0.606	0.693	0.299
9+	0.560	0.721	0.280
10+	0.450	0.756	0.205
11+	0.413	0.791	0.204
12+	0.367	0.818	0.185
13+	0.339	0.837	0.176
14+	0.303	0.860	0.162
15+	0.284	0.882	0.167
16+	0.248	0.904	0.152
17+	0.229	0.911	0.141
18+	0.220	0.925	0.145
19+	0.193	0.933	0.125
20+	0.193	0.939	0.132
21+	0.174	0.948	0.122
22+	0.147	0.951	0.098

Abbreviations: CADSS, Clinician-Administered Dissociative States Scale.

^*a*^Gray shading, 5+, indicates CADSS total score with the optimal discriminative properties, per the Youden Index (1950).

**Figure 1.**
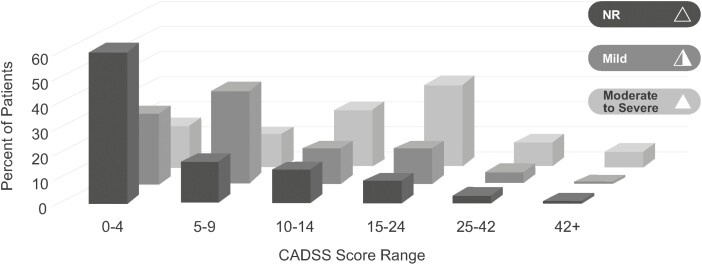
Distribution of CADSS total score by investigator-reported severity of dissociation. Abbreviations: CADSS, Clinician-Administered Dissociative States Scale; NR, adverse event of dissociation not reported. Note: The moderate and severe groups are combined due to the small number of patients rated as experiencing severe events.

Consistent with the results of the Youden Index, CADSS total scores generally followed the expected pattern, but with substantial variability (Figure 1). Specifically, as the severity of investigator-identified adverse events increased, the total CADSS score mean increased as well. However, the variability in scores was notable: in addition to poor specificity, only 37.2% (29/78) of the CADSS total scores of those for whom dissociation was identified as a mild adverse event fell in the 5–9 range. In contrast, most patients with reported moderate dissociation had CADSS total scores >9, and all 5 patients with reported severe dissociation had CADSS total scores >14.

### CADSS Factor Structure

According to CFA, neither the 3-factor solution reported by Niciu and colleagues (2018) nor the 1-factor solution reported by van Schalkwyk and colleagues (2018) provided a good fit to our CADSS data, based on the results of a goodness-of-fit test and goodness-of-fit based on skewed distribution. For the 1-factor model, only 1 of the 4 indices (SRMR) fell within the acceptable range (relative χ^2^ = 5.51, RMSEA = 0.80, SRMR = 0.05, and CFI = 0.85). Results for the 3-factor model were inconsistent as well, meeting criteria for acceptability on SRMR (0.05) and RMSEA (0.07) but not on relative χ^2^ (5.03) or CFI (0.87).

Principal axis factoring identified a 1-factor solution, with an eigenvalue of 8.5 and no other factor reaching 1.0. This single factor accounted for 86% of the variance, with 22 of the 23 items of the scale having loadings of at least 0.35. Given the single-factor solution, no rotation technique was required.

### CADSS Items Associated With Severity of Reported Dissociation Adverse Events

Table 3 depicts the CADSS items endorsed in each dissociation adverse event severity group. Items that more consistently increased in frequency and severity as reported adverse event severity increased centered around the themes of changes in bodily sensations, perceptual changes, and a general sense of being disconnected from one’s own experience (depersonalization). More unusual symptoms (e.g., having more than 1 identity) were less common. Notably, with the sole exception of “tunnel vision,” when moving from mild to moderate in dissociation adverse event severity, the percentage of patients with non-zero CADSS item severity ratings increased at least slightly for every item as the investigator-reported severity rating of dissociation increased. This is consistent with the unifactorial nature of the CADSS in this cohort.

**Table 3.** CADSS Item Endorsements by Severity of Dissociation as an Adverse Event as Reported by Investigators^*a*^

CADSS item	Not reported n = 655		Mild n = 78		Moderate n = 26	
	Patients with nonzero ratings, %	Mean severity	Patients with nonzero ratings, %	Mean severity	Patients with nonzero ratings, %	Mean severity
Things seem to be unreal	42.29	0.55	73.08	1.04	76.92	1.27
Things moving in slow motion	39.69	0.51	65.38	0.91	69.23	1.08
Body feels changed	27.63	0.35	56.41	0.85	61.54	1.19
Separation from what is happening	27.02	0.36	48.72	0.62	50	0.92
Watching situation as an observer	26.56	0.35	46.15	0.67	38.46	0.69
Spaced out, lost track	27.48	0.36	44.87	0.65	73.08	1.46
Disconnected from own body	29.16	0.37	42.31	0.62	57.69	1
Sounds disappeared or stronger	20.46	0.28	42.31	0.55	46.15	0.85
Things seem foggy and unclear	29.16	0.34	39.74	0.51	53.85	0.85
Looking from outside of your body	22.6	0.27	35.9	0.45	38.46	0.58
Interview longer than expected	16.95	0.2	30.77	0.36	42.31	0.62
Objects different than expected	18.47	0.24	25.64	0.38	26.92	0.46
Tunnel vision/wide-angle vision	16.34	0.2	24.36	0.32	23.08	0.38
Gaps in memory	14.96	0.19	23.08	0.37	38.46	0.65
Things cannot be accounted for	20	0.25	20.51	0.32	38.46	0.73
Colors diminished in intensity	13.13	0.15	17.95	0.26	30.77	0.46
Confused about who you really are	9.01	0.11	17.95	0.27	30.77	0.58
People seem dead, mechanical	9.92	0.13	14.1	0.19	23.08	0.38
Things happening very quickly	13.44	0.18	14.1	0.22	23.08	0.35
Color much brighter than expected	10.99	0.13	12.82	0.17	23.08	0.27
Parts of self do not fit together	10.38	0.13	12.82	0.24	19.23	0.38
More than 1 identity	4.12	0.05	12.82	0.17	19.23	0.27
Things very real, special clarity	14.35	0.19	11.54	0.15	19.23	0.23

Abbreviations: CADSS, Clinician-Administered Dissociative States Scale.

^*a*^Color coding of cells: At least 25% (blue), >33.3% (green), and >50% (orange) of patients received nonzero ratings. Results from patients with CADSS items rated as severe are not shown, owing to the small sample size (n = 5). CADSS items are rated on a scale from 0 (not at all) to 4 (severe); listed mean severity scores are the means of these values.

### Association Between Presence of Dissociation and Presence of Psychosis

The aforementioned infrequency of delusions and hallucinations being reported as adverse events limited our ability to quantitatively examine these adverse event reports other than to note that dissociation typically occurred without these phenomena. On the psychometric measures, Spearman rank correlation revealed a weak but statistically significant positive relationship between the CADSS and BPRS+ (*r* = 0.39, *P* < .01).

All 5 patients for whom hallucinations were reported had CADSS scores >4; however, 343 patients had CADSS scores >4 without reported hallucinations or delusions. Likewise, of the 113 patients for whom dissociation was reported as an adverse event, hallucinations were reported as an adverse event for only 3 (2.6%).

## DISCUSSION

Dissociation is a pleomorphic clinical construct used to describe a relatively heterogenous set of behavioral and sensory experiences in different clinical contexts. In the treatment of TRD with glutamate receptor modulators, the aspects of dissociation most commonly described as associated with treatment are more limited; however, substantial variability remains between the cutoffs used on the CADSS and the aspects of dissociation most likely to be identified and/or evoke concern from clinicians.

The results of our investigation of the characterization of dissociation in a group of patients with treatment-resistant MDD receiving their first treatment with esketamine nasal spray (in conjunction with recently started new oral antidepressant) echo this variability. We found that distinct dissociative experiences, as codified by the CADSS, did not cluster together but rather tended to move in the same direction in general as severity increased, consistent with the unifactorial nature of the CADSS items. Although the 4/5 threshold initially proposed by Bremner (2014) as an indicator for the presence of significant dissociation (primarily in patients with dissociative disorders) held up as the optimal cutoff for discriminating patients identified by adverse event report as experiencing dissociation, this cutoff was reasonably sensitive but not very specific. Correspondingly, the range of CADSS total scores seen at each level of reported adverse event severity is relatively broad, although the mean CADSS score increases with adverse event severity level as expected. We found that, as reported adverse event severity increases, CADSS items tend to increase quantitatively rather than qualitatively: specifically, the number of items endorsed increases, as do the item-level severity scores, as opposed to moderate-to-severe dissociation reflecting a different qualitative experience wherein the specific items endorsed change in a notable fashion. Finally, it is clear that dissociative experiences and positive psychotic symptoms, such as delusions and hallucinations, are not synonymous in our sample. This is consistent with recent work suggesting minimal overlap between dissociative and psychotic experiences (Piazza et al., 2022) as well as early work with ketamine as a model for schizophrenia, in which Krystal and colleagues (1994) noted that “hallucinatory behavior was limited to illusory experiences in all sensory domains, [with patients experiencing] alterations in perception of both self and environment.”

Some noteworthy differences exist between our study design and results compared with those reported by other groups investigating the factor structure of the CADSS among patients being treated with glutamatergic compounds. At the clinical level, both Niciu et al. (2018) and van Schalkwyk et al. (2018) were investigating i.v. ketamine, whereas our trial employed intranasal esketamine (the s-enantiomer of ketamine) in combination with a single oral antidepressant (duloxetine, escitalopram, sertraline, or venlafaxine extended-release). Furthermore, our sample was composed entirely of patients with strictly defined treatment-resistant MDD; in contrast, while the samples of Niciu et al. (2018) and van Schalkwyk et al. (2018) were primarily composed of treatment-resistant MDD, both samples also included patients with depression associated with bipolar disorder. Regarding statistical power, our analyses were conducted in a much larger sample size (n = 764) than those of either Niciu et al. (2018) or van Schalkwyk et al. (2018) (n = 126 and 110, respectively). Given the relatively large ratio of items to factors being investigated (23/3 = 7.67) and the low-to-moderate levels of communality observed in these types of data, the large sample assessed in our analysis would be expected to provide more robust factor estimates than those obtained in sample sizes close to 100 (MacCallum et al., 1999; Osborne and Costello., 2004).

The modest correlation between investigator report and standardized assessment is not unique to this treatment setting (Cuijpers et al., 2010; Dang et al., 2020). Adverse event reports and formal measures are inherently calibrated to different standards: clinicians were encouraged to report any adverse event they believed was clinically relevant or merited treatment, whereas the solicited formal measures assessed at each treatment session (e.g., CADSS, BPRS+) aim to detect deviations from an accepted standard of “normal” values. Therefore, higher rates are more often revealed by the formal measures obtained by solicited reporting than by spontaneous clinician reporting. A potential, relatively understudied contributor to this inconsistency is the variability (or lack thereof) in the “trigger point” aspects of patient presentation (e.g., presence or severity of specific symptoms) at which different clinicians will report the presence of an adverse event. We attempted to control for this variability source to some extent by using only the data from the first treatment session; thus, we removed the influence of any patient-specific adjustments that might occur after the first session, wherein (for instance) patient or clinician could judge based on previous sessions whether a given symptom was likely to be clinically relevant. However, this would not account for a priori differences in patient/clinician perspectives on which patterns/level of severity of dissociative symptoms met “clinically relevant” criteria. Notably, another reason for assessing dissociation symptoms at the first treatment session is that the severity level of this adverse event as rated by the CADSS typically appears highest at the first treatment session and then attenuates across subsequent sessions, despite persistence of the antidepressant effect (Chen et al., 2022).

There was no single CADSS total score cutoff that could be viewed as a *strong* indicator of dissociation identified as an adverse event. Other groups have noted that the CADSS was not designed for this purpose (e.g., van Schalkwyk et al., 2018). Because the use of the CADSS as a structured tool in investigations of glutamate receptor modulators is nearly ubiquitous (Rodrigues et al., 2021), it does have some value in helping quantify the dissociative experience associated with these medications and in comparing results across studies. However, it may be more informative to consider the CADSS total score as a measure of how the dissociative experiences change over time rather than as an indicator of the presence or absence of a level of dissociation that an observing clinician would view as clinically significant.

Item analysis across the range of reported adverse event severity suggests that there is no “universal” profile of dissociative symptoms associated with esketamine nasal spray. However, we found that, similar (but not identical) to the symptom clusters noted by van Schalkwyk and colleagues (2018), changes in bodily sensations, general perceptual changes, and a general sense of being disconnected from one’s own experience (depersonalization) increase in frequency as the severity of investigator-reported dissociation adverse events increases. Interestingly, there is substantial overlap between the CADSS items that are most likely to be endorsed and those identified by Rodrigues and colleagues (2021) in their proposed 6-item CADSS short form. In our sample, 10 items were endorsed by at least one-third of patients for whom mild dissociation was reported — 5 out of the 6 items on the short form of Rodrigues et al. (2021) (all except “gaps in memory”) are included within these 10. Notably, these same 5 items were reported as present in at least 25% of patients for whom dissociation was not reported as an adverse event. The cross-sample consistency lends some confidence to the notion that, even with the evident variability, some CADSS items may be commonly associated with dissociative experiences among patients treated with i.v. ketamine or esketamine.

In contrast, the relationship between dissociation and positive symptoms of psychosis is weak, unlike the pattern noted among clinical and nonclinical samples in a recent meta-analysis of studies that predominantly included participants suffering from psychotic disorders (Longden et al, 2020). Within the context of this treatment, this relationship is affected in large part by which specific aspects of psychological experience one considers to be a positive symptom of psychosis. Specifically, in line with the early work describing the effects of ketamine (e.g., Oye et al., 1992; Krystal et al., 1994), treatment-associated hallucinations and delusions are rare. However, treatment-associated illusions and perceptual alterations of the self and environment, which overlap to some extent with the boundaries of the construct of dissociation as instantiated by the CADSS, occur more frequently and are likely the source of the weak but statistically significant positive relationship between the CADSS and BPRS+. Clarification of these concepts is critical to understanding the comments of some authors suggesting that psychotic symptomatology is not uncommon during the treatment of depression with glutamate receptor modulators (Tashakkori et al., 2021). This distinction is important for both the sake of informing patients of their potential experience as well as to understanding potential mechanisms of action.

### Limitations

As noted, these data are limited to those collected during the first (to our knowledge) open-label treatment session for patients being treated for TRD using esketamine nasal spray; consequently, doses were limited to 28 mg in patients ≥65 years and 56 mg for all others (Wajs et al., 2020). Reporting of adverse events related to dissociation occurred according to the clinical judgment of the investigator, and the protocol did not provide any specific instructions beyond general adverse events reporting guidelines. The extent to which these findings may vary by diagnosis, age, gender, time in treatment, baseline level of symptomatology, or interactions thereof remain unexplored. The extent to which the reporting of these adverse events was affected by the knowledge that the CADSS and BPRS were being quantified is also unknown.

In summary, although adverse event reports and CADSS scores generally move in the same direction, there is substantial variability in the dissociation-related experiences of patients taking esketamine, and there appears to be similar variability in the correlation between CADSS total scores and the identification of dissociation as an adverse event by observing clinicians. Even with this variability, however, it is clear that overt psychotic symptoms (delusions or hallucinations) are not a common aspect of the dissociative experiences associated with esketamine nasal spray administered in the dose range and frequency for which this agent is approved for use in TRD.

## Data Sharing and Accessibility

The data sharing policy of Janssen Pharmaceutical Companies of Johnson & Johnson is available at https://www.janssen.com/clinical-trials/transparency. Requests for access to the study data can be submitted through Yale Open Data Access (YODA) Project site at http://yoda.yale.edu.

## Interest Statement

Drs David Williamson, Ibrahim Turkoz, Stephane Borentain, Ewa Wajs, Jaskaran B. Singh, and Wayne C. Drevets were employees of Janssen Scientific Affairs, Janssen Research & Development, LLC, or Janssen Research & Development Belgium at the time this work was conducted and hold company equity.

## Previous Presentations

Elements of this research were presented in poster form at the 15th Annual Scientific Meeting of the International Society for CNS Clinical Trials and Methodology, February 19-21, 2019, Washington, DC.
